# Impact of prosthesis-patient mismatch after mitral valve replacement in rheumatic population: Does mitral position prosthesis-patient mismatch really exist?

**DOI:** 10.1186/s13019-017-0653-x

**Published:** 2017-10-10

**Authors:** Seung Hyun Lee, Byung Chul Chang, Young-Nam Youn, Hyun Chel Joo, Kyung-Jong Yoo, Sak Lee

**Affiliations:** Division of Thoracic and Cardiovascular Surgery, Severance Cardiovascular Hospital, Yonsei Cardiovascular Research Institute, Yonsei University, College of Medicine, 250 Seongsanno, Seodaemun-gu, Seoul, 03722 Republic of Korea

**Keywords:** Heart valve prosthesis, Hemodynamics, Mitral valve, Mortality, Surgery, Valves

## Abstract

**Background:**

Prosthesis-patient mismatch (PPM) is characterised by the effects of inadequate prosthesis size relative to body surface area (BSA).The purpose of this study was to determine the impact of PPM on late clinical outcomes after mitral valve replacement (MVR) in rheumatic population.

**Methods:**

From 2000 to 2013, a total of 445 patients (mean age 54.2 ± 11.7 years) underwent isolated MVR (±tricuspid annuloplasty) for rheumatic disease were investigated. Effective orifice area (EOA) was determined by the continuity equation and PPM was defined as indexed EOA (EOA/BSA) ≤ 1.2 cm^2^/m^2^. Clinical and echocardiographic follow-up (mean follow up 8.7 ± 4.0 years) results were compared.

**Results:**

37% of patients (*n* = 165) had PPM. There were no significant differences in baseline and operative characteristics between patients with and without PPM except age and IEOA. A significant decrease in mean trans-valvular pressure gradient (MPG) over time following MVR, however the change of MPG showed no differences between groups (No PPM vs. PPM: 8.9 ± 4.7 mmHg → 3.6 ± 1.2 mmHg vs. 8.7 ± 4.5 mmHg → 3.8 ± 1.4 mmHg, *p*-value = 0.28). In all patients, there was a reduction of left atrium dimension (58.6 ± 12.0 mm → 53.2 ± 12.0 mm vs. 57.9 ± 8.9 mm → 52.2 ± 8.9 mm, *p*-value = 0.68) and left ventricular end diastolic diameter (49.9 ± 5.7 mm → 48.9 ± 5.7 mm vs. 49.7 ± 6.0 mm → 48.3 ± 5.0 mm, *p* = 0.24) without statistical significance. Freedom from TR progression rates at 3 and 5 years (99% vs.98%, 99% vs. 98%, *p*-value = 0.1), and overall survival rates at 3 and 5 years (97% vs. 96%, 94% vs. 94%, *p*-value = 0.7) were similar.

**Conclusion:**

This study shows that mitral PPM is not associated with atrial /ventricular remodeling and might not influence late clinical outcome including late TR progression, survival in rheumatic population.

## Background

Previous studies reported that prosthesis–patient mismatch (PPM) in aortic valve position is strongly associated with worse hemodynamics, less regression of left ventricular hypertrophy, more cardiac events, and higher mortality rates after aortic valve replacement [1–3]. However, PPM following mitral valve replacement (MVR) has been still less investigated. Previous studies reported that mitral PPM is various, from 30 to 85% when in vivo evaluation of effective orifice area (EOA) is performed [4–6]. Some studies on the clinical impact of PPM following MVR on survival have reported conflicting results, although two recent trials showed that PPM in the mitral position independently affects long-term survival.

Dumesnil et al. have addressed that indexed EOA derived from in vivo postoperative measures is the only parameter that can consistently be correlated with postoperative gradients as well as clinical outcomes in defining PPM [7]. Mitral PPM can be equated to residual mitral stenosis resulting in increased trans-mitral gradients, increased left atrial pressure, and pulmonary hypertension (PH). These factors may lead to right ventricular dilatation/dysfunction and to atrial fibrillation, which may, in turn, lead to tricuspid annulus dilatation and functional tricuspid regurgitation (fTR).

However, despites of small sized mitral prosthesis implantation such as 25 mm, we can easily find that no occurrence of TR after MVR during follow duration. In Asian rheumatic population, mitral annuls size is relatively small compared with western mitral disease, so we can easily meet the patients with small sized mitral prosthesis replacement and collect many cases. The objective of this study was to investigate the impact of mitral PPM on late clinical outcomes including TR progression and survival following MVR in rheumatic population.

## Methods

### Patient population

We retrospectively reviewed a consecutive series of 445 patients who underwent elective isolated MVR with or without TAP (tricuspid annuloplasty) for rheumatic mitral valve disease at Severance Cardiovascular Hospital, University of Yonsei, from Jan 2000 to Dec 2013. Patients with concomitant aortic valve, coronary artery bypass and aorta surgery were excluded. In cases of TAP, if residual TR grade after TAP were 2,3 and 4, they were also excluded for minimizing the confusion of TR progression analysis. Data were retroprospectively collected and recorded in an electronic database, and clinical follow-up was completed with routine outpatient clinics. Patients who did not present at the visit were contacted by telephone, and all symptoms, mortality, and any complications that occurred during follow-up were recorded. This study was approved by the Institutional Review Board of Yonsei University College of Medicine. Individual patient consent was waived because this study did not interfere with patient treatment, and the database was designed so that individual patients could not be identified. All baseline and clinical characteristics were obtained from the medical record of patients.

All patients underwent a full median sternotomy and operation performed on cardio-pulmonary bypass. The prostheses were used in as followings:

- Mechanical prosthesis: St. Jude Medical Standard Mechanical (St. Jude Medical Inc., St. Paul, MN) (*n* = 140), MIRA (Edwards Lifesciences; Irvine, Calif) (*n* = 54), ATS (Medtronic, Minneapolis, MN) (*n* = 85), Sorin (Sorin Biomedica, Saluggia, Italy) (*n* = 11), ON-X (On-X Life Technology, Austin, TX) (*n* = 61), CarboMedics Mechanical (Sulzer CarboMedics, Austin, TX) (*n* = 12).
- Bioprosthesis: Perimount Magna (Edwards Lifesciences LLC, Irvine, CA) (*n* = 49), Epic (St. Jude Medical Inc., St. Paul, MN) (*n* = 17), Hancock (Medtronic, Minneapolis, MN) (*n* = 7), Biocor (St. Jude Medical Inc., St. Paul, MN) (*n* = 11). In all cases, posterior chordal preservation was attempted as a routine maneuver.

### Doppler-echocardiographic assessment

Clinical and echocardiographic assessment was performed prior to MVR and 12–60 months after operation. The echocardiographic images of the included patients were reanalyzed by 2 experienced echocardiographers who were unaware of the patient’s clinical data. LV internal diameter, septal thickness, and LV posterior wall thickness were measured at end-diastole. LV mass was calculated using the formula developed by Devereux et al. [8], and LV mass was indexed for the body surface area. The left atrial volume was calculated from the parasternal long-axis view and apical four-chamber view using the prolate ellipse method [9]. The severity of TR was assessed using color flow imaging and regurgitant jet area [10]. Doppler color flow mapping was used to assess the competency of the prosthetic valves.

### EOA calculation and definition of PPM

The in-vivo prosthetic valve effective orifice area (EOA) was calculated with the use of the continuity equation, using the stroke volume measured in the LV outflow tract divided by the integral of themitral valve transprosthetic velocity-time integral during diastole. The indexed EOA (IEOA) was calculated by dividing the measured EOA by the patient’s body surface area (BSA) at the time of follow-up. Indexed EOA was used to define PPM as significant if ≤ 1.2 cm^2^/m^2^, moderate if > 0.9 cm^2^/m^2^ and ≤ 1.2 cm^2^/m^2^, and severe if ≤ 0.9 cm^2^/m^2^ [4].

### Statistical analysis

Data were prospectively collected and recorded in an electronic database; statistical analysis was performed using the Statistical Package for the Social Sciences, version 11.0 (SPSS, Chicago, IL). Continuous data are expressed as the mean and standard deviation; categorical data are expressed as the percentage, comparisons were made using the 2-sample t and the χ2 or the Fischer exact tests, respectively. We gain the optimal cutoff value of IEOA for late TR progression and mortality, receiver operating curve (ROC) method was used. Comparison between group with or without PPM, Kaplan Meyer survival analysis was used and *p* value < 0.05 was considered statistically significant.

## Results

### Mitral valve patient–prosthesis mismatch

Of the 445 study patients, PPM was in 165 (37.1%), severe in only 8 by the definition of PPM. The proportion of patients with PPM was lower in those with mechanical valves (*n* = 362) than those with bioprosthesis (*n* = 83) (*n* = 116, 32% vs. *n* = 49, 59%, *p* < 0 .01). An IEOA of patients with mechanical valves had a higher than those with bioprosthesis (1.41 ± 0.31 vs. 1.15 ± 0.21, *p* < 0.01). The clinical characteristics of the patients are shown in Table 1. Female portion of patients with PPM were similar (52, 31.5% vs. 69, 24.6%, *p* = 0.09) and PMV (Percutaneous mitral valvuloplasty) history were also not significantly different between groups (37, 8.3% vs. 66, 14.8%, *p* = 0.8). However, the age (55.96 ± 12.36 vs. 53.28 ± 10.82, *p* < 0.01) and BSA (body surface area, m^2^) in PPM group (1.61 ± 0.14 vs.1.55 ± 0.15, *p* < 0.01) was significantly higher than no PPM group regardless gender. Tissue valve portion was meaningfully higher in PPM group compared with No PPM group (29.7% vs. 12.1%).

**Table 1:** Basic preoperative charateristics

Preoperative parameters			
	PPM (*n* = 165)	No PPM (*n* = 280)	*p* value
Age (years)	55.96 ± 12.36	53.28 ± 10.82	0.02
Gender (Female, n, %)	52 (31.5%)	69 (24.6%)	0.09
BSA (cm/m^2^)	1.61 ± 0.14	1.55 ± 0.15	< 0.01
Valve type (Tissue No, %)	49 (29.7%)	34 (12.1%)	< 0.01
Previous PMV Hx. (n, %)	37 (8.3%)	66 (14.8%)	0.82
LVEF (%)	62.70 ± 9.28	60.71 ± 9.52	0.31
LVEDD (mm)	48.27 ± 5.06	48.98 ± 5.72	0.23
LVESD (mm)	32.98 ± 5.17	33.8 ± 6.25	0.19
LAD (AP, mm)	52.29 ± 8.98	53.42	0.38
MPG (mmHg)	8.7 ± 4.52	8.91 ± 4.71	0.38

*BSA* body surface area, *PMV* percutaneous mitral valvuloplasty, *LVEF* left ventricular ejection fraction, *LVEDD* left ventricular end diastolic dimension, *LVESD* left ventricular end systolic dimension, *MPG* mean pressure gradient

### Perioperative data and early clinical outcomes

Perioperative and early outcomes including postoperative echocardiography data are shown in Table 2. Thirty-day mortality was similar between groups (0% vs. 0.2%, *p* = 1.0), and postoperative CVA (cerebrovascular accidents) was also not different (0% vs. 0.2%, *p* = 1.0). There were also similar rates of other morbidities (postoperative bleeding: 1.8% vs. 1.1%, *p* = 0.08, acute renal failure: 0.2% vs. 0.7%) between groups.

**Table 2:** Early clinical outcomes and the the change of hemodynamics parameters

Perioperative and postoperative parameters			
	PPM (*n* = 165)	No PPM (*n* = 280)	*p* value
30 days mortlaity	0 (0%)	1 (0.2%)	1.0
CVA (n, %)	0 (0%)	1 (0.2%)	1.0
ARF (n,%)	1 (0.2%)	3 (0.7%)	1.0
Postoperative Bleeding (n,%)	8 (1.8%)	5 (1.1%)	0.08
Prosthesis size (mm)	27.58 ± 1.57	27.89 ± 1.55	0.03
EOA (cm^2^)	1.69 ± 0.15	2.38 ± 0.41	< 0.01
IEOA	1.06 ± 0.08	1.55 ± 0.25	< 0.01
The change of RVSP (mmHg)	10.85 ± 14.75	9.91 ± 14.75	0.53
The change of MPG (mmHg)	4.7 ± 4.5	5.3 ± 5.0	0.34

*CVA* cerebral vascular event, *ARF* acute renal failure, *EOA* effective orifice area, *IEOA* indexed effective orifice area, *RVSP* right ventricular systolic pressure, *MPG* mean pressure gradient

The mean prosthesis size (mm) in No PPM group was significantly bigger than PPM group (27.89 ± 1.55 vs. 27.58 ± 1.57, *p* = 0.03). Values of EOA (2.38 ± 0.41 vs. 1.69 ± 0.15, *p* < 0.01) and IEOA (1.55 ± 0.25 vs.1.06 ± 0.08, *p* < 0.01) in No PPM group were also siginificantly bigger than PPM group (Table 2). The ealry change of MPG (mmHg) (4.7 ± 4.5 vs.5.3 ± 5.0, *p* = 0.34) and right ventricular systolic pressure (RVSP, mmHg) (10.85 ± 14.75 vs.9.91 ± 14.75, *p* = 0.53) after MVR was similar between groups.

### Late clinical outcomes including postoperative echocardiography data, TR progression and survival

Mean echocardiography follow up duration was 8.4 ± 3.7 (0.3–15.2) years. Left ventricular ejection fraction (LVEF, %), left ventricular end systolic dimension (LVESD, mm), left ventricular end systolic dimension (LVEDD, mm), left atrim size (anterior-posterior distance, mm), and mean pressure gradient (MPG, mmHg) were routinely checked during follow up for analyzing ventricular remodeling. LVEF improvement in No PPM group was significantly better than PPM group (Δ: 0.83 ± 13.77 vs. 0.98 ± 9.70, *p* = 0.89). Left atrium (Δ LAD: 11.0 ± 18.8 vs.13.5 ± 19.1, *p* = 0.19) and ventricular remodeling (Δ LVESD: 5.6 ± 12.4 vs. 4.1 ± 11.7, *p* = 0.2 and Δ LVEDD: 8.3 ± 17.8 vs.5.7 ± 16.0, *p* = 0.13) were similar between groups. The reduction of right ventricular systolic pressure (RVSP, mmHg) (8.75 ± 8.2 vs.10.7 ± 17.9 *p* = 0.30) and the MPG change (4.9 ± 4.5 vs. 5.3 ± 5.0, *p* = 0.34) were also not siginicantly different between groups (Table 3).

**Table 3:** Serial change of hemodynamic characteristic and remodelling data

		Preoperative	Immediate postoperative	Last followup
LVEF (%)	No PPM	60.71	60.30	62.09
	PPM	61.70	61.60	62.68
LVESD (mm)	No PPM	34.64	34.21	33.48
	PPM	33.76	33.08	33.09
LVEDD (mm)	No PPM	49.92	48.67	48.69
	PPM	49.30	47.94	48.06
LAD (AP, mm)	No PPM	58.12	51.32	52.62
	PPM	57.88	51.03	52.17
MPG (mmHg)	No PPM	8.99	3.59	3.76
	PPM	8.83	4.01	4.02
RVSP (mmHg)	No PPM	42.33	31.82	32.96
	PPM	43.75	32.56	33.02

*LVEF* left ventricular ejection fraction, *LVEDD* left ventricular end diastolic dimension, *LVESD* left ventricular end systolic dimension, *LAD* left atrium dimension, *MPG* mean pressure gradient, *RVSP* right ventricular systolic pressure

**Fig. 1:**
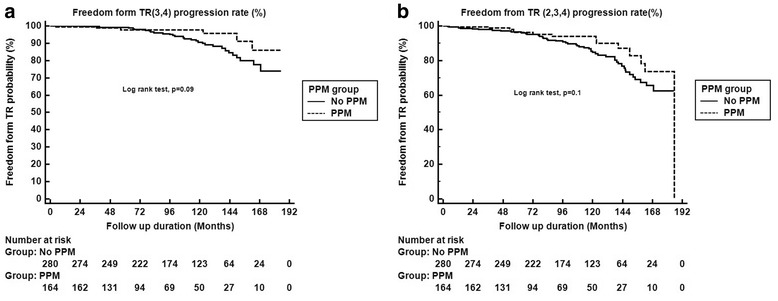
Freedom from TR progression rate at 3, 5, and 10 years between groups (PPM vs. No PPM). **a** Freedom from TR progression (3,4) rate at 3, 5, and 10 years was 99%, 98%, and 93%, respectively. Freedom from TR progression (3,4) rate in patients with PPM at 3, 5, and 10 years was similar to that of patients without PPM (98%, 98%, 98% vs. 99%, 99%, 91%, respectively, *P* = 0.09). **b** Freedom from TR progression (2,3,4) in patients with PPM at 3, 5, and 10 years was similar to that of patients without PPM (98%, 96%, 94% vs. 98%, 96%, 85%, respectively, *P* = 0.10)

Freedom from TR progression (3,4) rate at 3, 5, and 10 years was 99%, 98%, and 93%, respectively. Freedom from TR progression (3,4) rate in patients with PPM at 3, 5, and 10 years was similar to that of patients without PPM (98%, 98%, 98% vs. 99%, 99%, 91%, respectively, *P* = 0.09) (Fig. 1a). After including TR grade 2, freedom from TR progression (2,3,4) in patients with PPM at 3, 5, and 10 years was similar to that of patients without PPM (98%, 96%, 94% vs. 98%, 96%, 85%, respectively, *P* = 0.10) (Fig. 1b).

**Fig. 2:**
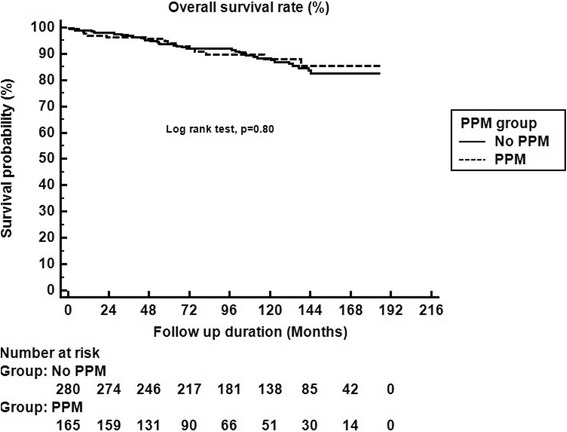
Overall 3, 5, and 10-year survivals were 97%, 94%, and 88%, respectively. Patients with PPM had similar 3, 5, and 10-year survivals compared with no PPM patients (96%, 94% and 88% vs. 97%, 94% and 88%, respectively, *P* = 0.80)

Overall 3, 5, and 10-year survivals were 97%, 94%, and 88%, respectively. Patients with PPM had similar 3, 5, and 10-year survivals compared with no PPM patients (96%, 94% and 88% vs. 97%, 94% and 88%, respectively, *P* = 0.80) (Fig. 2).

Sub-analysis of severe PPM patients was as followings: Total eight patients had severe PPM (definition: IEOA ≤ 0.9 cm2/m2) and average age was 62.63 years (37 ~ 71). BSA was 1.7 (1.56 ~ 1.81) and preoperative TR were all under mild (Gr1). EF was 58.5% (36 ~ 71%), LVEDD was 52.5 mm (47 ~ 59 mm) and LA size (AP diameter, mm) was 59.3 mm (51 ~ 65 mm). Average IEOA was 0.85 (0.82~0.88). Five were Hancock (*n* = 2/27 mm, *n* = 3/29 mm, Medtronic, Minneapolis, MN), 2 were Epic (*n* = 2/29 mm, St. Jude Medical Inc., St. Paul, MN) and 1 was Perimount Magna (*n* = 1/25 mm, Edwards Lifesciences LLC, Irvine, CA). There was no in-hospital mortality and postoperative newly onset TR was none in all cases. There was just one case of late mortality after postoperative 6 years due to stomach cancer, but this was not correlated with cardiac death or TR progression.

**Fig. 3:**
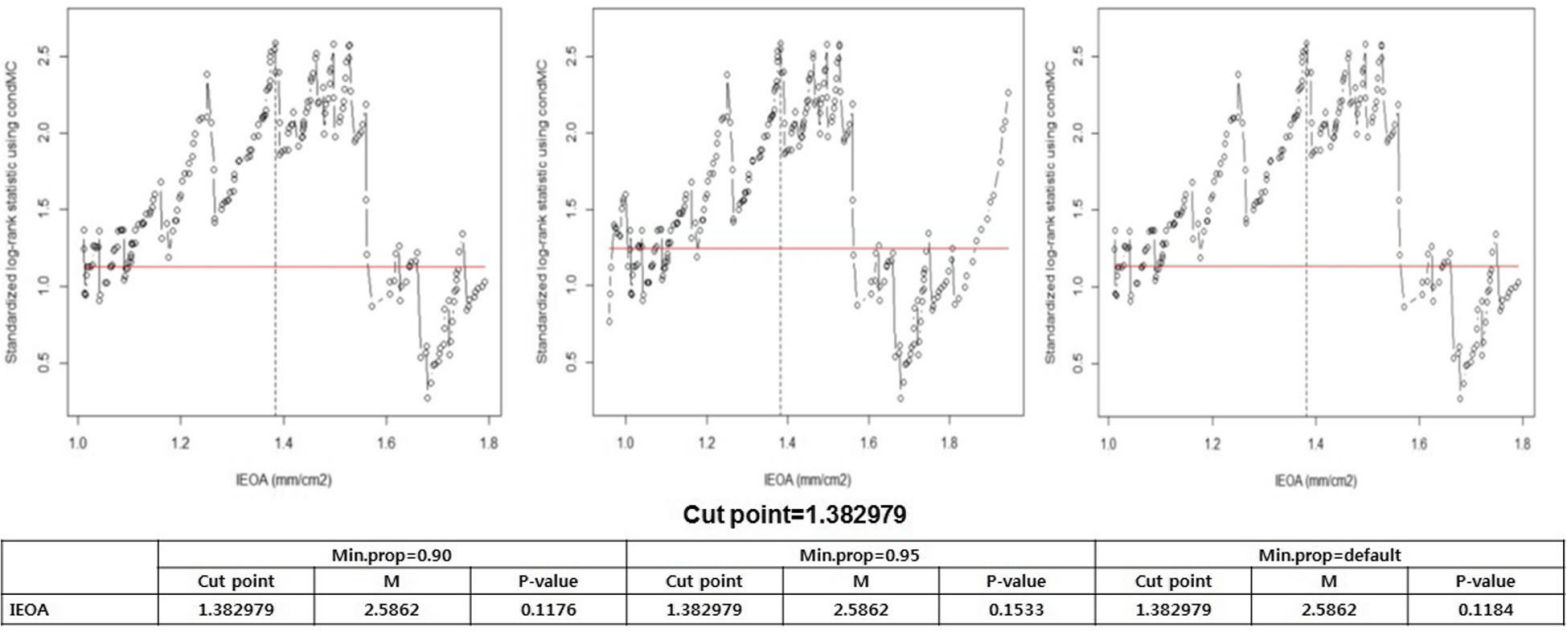
ROC method for the gaining optimal IEOA cut value of TR progression 1.38 was determined as a cut value (new PPM value) for TR progression

**Fig. 4:**
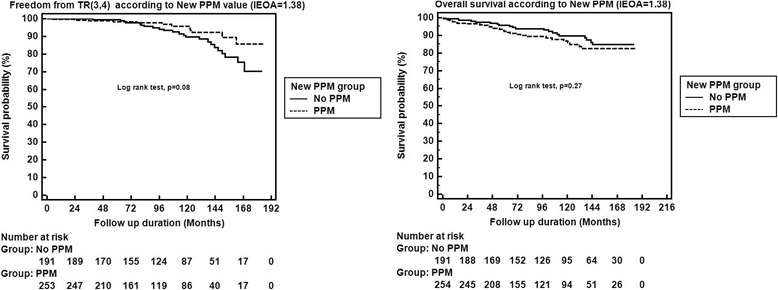
Freedom from TR progression and overall survival at 3,5 and 10 years. **a** Freedom from TR progression at 3,5 and 10 years (99, 99% and 88% vs. 99%, 98% and 97%, respectively, *P* = 0.02). **b** Freedom from all cause mortality at 3, 5, and 10-year (96%, 92% and 86% vs. 97%, 96% and 89%, respectively, *P* = 0.16)

For the gaining optimal IEOA cut value of TR progression, we used ROC method and 1.38 was determined as a cut value (new PPM value) for TR progression (Fig. 3). We divided two groups based on 1.38 (IEOA, new PPM) and compared late TR progression and cumulative survival. But against our expectation, the larger IEOA group (1.39 ≤, No PPM) showed inferior tendency of freedom from TR progression at 3,5 and 10 years (99, 99% and 88% vs. 99%, 98% and 97%, respectively, *P* = 0.08) (Fig. 4a). For survival, Patients with PPM had similar 3, 5, and 10-year survivals compared with no PPM patients (96%, 92% and 86% vs. 97%, 96% and 89%, respectively, *P* = 0.16) (Fig. 4b).

Considering valve type such as mechanical and tissue, late bleeding event related with warfarin intake showed higher in mechanical prosthesis group (*n* = 11, 3.1%) compared with tissue prosthesis (*n* = 2, 2.4%) as expected. Late CVA incidence was 6.3% in mechanical and 10.8% in tissue prosthesis group. Reoperation related with degeneration was 1.1% in mechanical and 3.6% in tissue prosthesis group.

## Discussion

The main findings of the present study are as followings: (1) the incidence of PPM after MVR in patients with rheumatic mitral stenosis was relatively high against our expectation; (2) mitral position PPM was not correlated with late clinical result including late TR progression and survival; (3) the larger IEOA, the more TR progression tendency (*p* = 0.08), ironically.

Since first described in 1978 [11], PPM after MVR has been suggested to be correlate with poor clinical outcomes including persistent pulmonary hypertension and late functional tricuspid regurgitation [2, 12]. Until now, well descripted mechanism of the adverse effect of PPM was originated from high residual transvalvular pressure gradients, which is same with residual mitral stenosis with similar consequences (ie, the persistence of abnormally high mitral gradients and increased left atrial and pulmonary arterial pressures). The major consequence of pulmonary hypertension is right ventricular failure, which generally results from chronic pressure overload and associated volume overload with the development of tricuspid regurgitation. Hence, the persistence of left atrial and pulmonary hypertension associated with PPM is likely the main factor responsible for the increased mortality observed in the patients with severe PPM [4].

However, some reports suggested that PPM did not affect survival after MVR [13, 14], although several recent trials showed that mitral PPM independently affects long-term survival [4, 5]. Mitral valve replacement itself should ideally improve pulmonary hypertension and tricuspid regurgitation without increasing operative times and risk. The negative effects of aortic valve PPM on left ventricular remodeling, functional status, early mortality, and late survival have been extensively corroborated. [1, 15–17] What remains uncertain is whether clinically deleterious effects of PPM could be encountered after MVR.

Actually, there has been a recent rising interest in mitral PPM, which has been well documented in the pediatric population from small BSA and limited size of prosthesis according to growth potential. A variety of factors have been used in an attempt to define the concept of clinical PPM in children: These have included size/weight ratios, Z scores, maximum transprosthesis velocity (Vmax), and 2.5 times increase in body weight from the time of implant. In all instances, these factors have been correlated with outcomes (early mortality, survival, and PTH) [18, 19]. Adult mitral PPM was the subject of an original case report in 1981 [20], it has subsequently been theorized, through in vitro pulse duplicator analysis, that an indexed geometrical area (IGOA) less than 1.3 to 1.5 cm2/m2 could potentially leave the patient with high postoperative trans-mitral gradients. In a clinical study, a good correlation between elevated transprosthetic mitral gradient and in vivo IEOA was demonstrated by the use of the continuity eq. (CE) during echocardiographic assessment of porcine mitral prostheses [7]. In this report, an IEOA of 1.3 to 1.5 cm2/m2 or less at rest was associated with a mean mitral gradient of 4 mmHg; with every 10% increase in stroke volume (maximum 50%), there was a proportional increase of the mean mitral gradient.

**Fig. 5:**
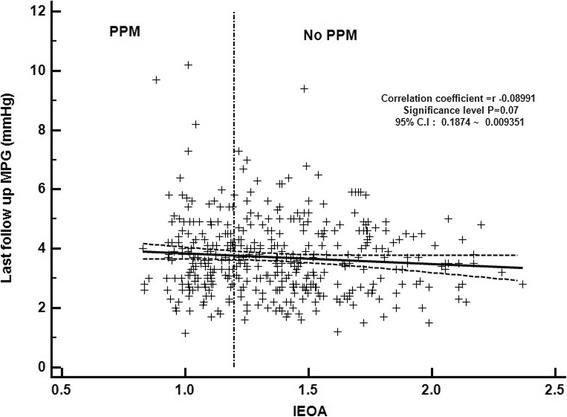
Correlation between trans-mitral gradients and IEOA induced from correlation coefficient analysis (*r* = − 0.08, *p* = 0.07)

**Fig. 6:**
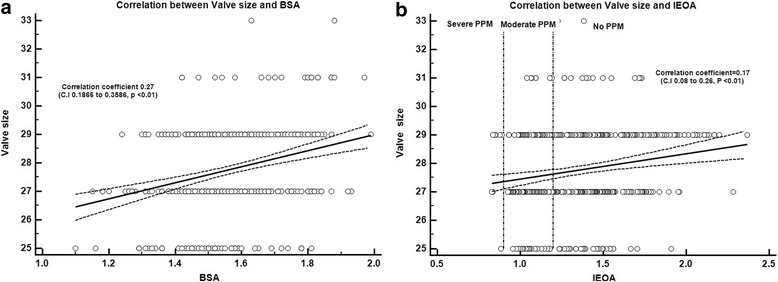
Correlation between valve implantation size and BSA, IEOA. **a** Correlation between valve implantation size and BSA (*r* = 0.27, *p* < 0.01). **b** Correlation between valve implantation size and IEOA (*r* = 0.17, *p* < 0.01)

However, in our study, there was very weak relation between trans-mitral gradients and IEOA induced from correlation coefficient analysis (*r* = − 0.08, *p* = 0.07) (Fig. 5). It means that postoperative decrease of MPG was induced well regardless of IEOA and there was no problematic residual pressure gradients which can make physiologic mitral stenosis (Fig 6a). Also we found that BSA did not show strong correlation with IOEA (*r* = 0.27) and PPM incidence was not significantly different under 29 mm sized population using same correlation coefficient analysis (*r* = 0.17) (Fig. 6b). In asian population, small size mitral implantation might be considered generally to be occured more frequently rather than western population, however implantation prosthesis size was not so strongly associated with BSA. Mitral prosthesis size in No PPM group was slightly bigger than PPM group (27.89 ± 1.55 vs. 27.58 ± 1.57, *p* = 0.03), but after exclusion of 31 mm size implanted patients, there was no significant different between groups (27.44 ± 1.42 vs. 27.69 ± 1.35, *p* = 0.08). From the analysis of TR progression rate, we found contradictory result that PPM group showed superior freedom from TR progression rate in both classic IEOA classficaiton (1.25) and newly gained IEOA (1.38) classification. This doesn’t necessarily mean that the bigger mitral prosthesis, the better clinical outcomes. Bigger mitral implantation might mean two things: 1) Big BSA or 2) Heart enlargement, however, from our result, big BSA doesn’t necessarily mean bigger mitral implantation (Fig. 6a, *r* = 0.27). Then mitral annulus enlargement from heart failure might be strong candidate for bigger prosthesis implantation. Actually mitral annulus enlargement sometimes closely connected with heart dysfunction and TR can be more frequently progressed in this group. Recently published study about mitral PPM suggested mitral PPM significantly affects longterm outcomes after mitral valve replacement in terms of long-term survival and freedom from cardiac death [21], however this study was just analyzed by reference values and very limited old types of valves.

## Study limitations

Our study has limitations that must be recognized. First, this study was a retrospective study, although all consecutive patients who underwent MVR for rheumatic population during the study period were enrolled. Second, this study was limited in rheumatic population in order to increase the possibility of mitral PPM under hypothesis that rheumatic mitral stenosis actulally showed small mitral annulus compared with other mitral pathologies. Threfore, further evaluation for the extension of population should be needed.

## Conclusions

PPM is not an independent predictor of late onset TR progression and long term survival rate after MVR. Hemodynamic positive change and remodeling were similar regardless of PPM. Also contrary to expectations, BSA was not so strongly correlated with EOA and the bigger IEOA showed close correlation with TR progression tendency. So we suggest that mitral PPM might have not clinical significance in real world and the most important thing in MVR is safe implantation by appropriate sizing.

## Abbreviations

ARF: Acute renal failure
BSA: Body surface area
CE: Continuity equation
CVA: Cerebral vascular event
EOA: Effective orifice area
fTR: Functional tricuspid regurgitation
IGOA: Indexed geometrical area
LVEDD: Left ventricular end diastolic dimension
LVEF: Left ventricular ejection fraction
LVESD: Left ventricular end systolic dimension
MPG: Mean pressure gradient
MPG: Mean trans-valvular pressure gradient
MVR: Mitral valve replacement
PH: Pulmonary hypertension
PMV: Percutaneous mitral valvuloplasty
PPM: Prosthesis-patient mismatch
ROC: Receiver operating curve
RVSP: Right ventricular systolic pressure
TAP: Tricuspid annuloplasty
